# Use of a bovine genome array to identify new biological pathways for beef marbling in Hanwoo (*Korean Cattle*)

**DOI:** 10.1186/1471-2164-11-623

**Published:** 2010-11-09

**Authors:** Seung-Hwan Lee, Cedric Gondro, Julius van der Werf, Nam-Kuk Kim, Da-jeong Lim, Eung-Woo Park, Sung-Jong Oh, John P Gibson, John M Thompson

**Affiliations:** 1 Animal Genomics & Bioinformatics Division, National Institute of Animal Science, RDA, Suwon 441-706, Korea; 2 School of Environmental and Rural Science, University of New England, Armidale, NSW 2351, Australia; 3 The Cooperative Research Centre for Beef Genetic Technologies, University of New England, Armidale, NSW 2351, Australia; 4 The Centre for Genetic Analysis and Applications, University of New England, Armidale, NSW 2351, Australia

## Abstract

**Background:**

Marbling (intramuscular fat) is a valuable trait that impacts on meat quality and an important factor determining price of beef in the Korean beef market. Animals that are destined for this high marbling market are fed a high concentrate ration for approximately 30 months in the Korean finishing farms. However, this feeding strategy leads to inefficiencies and excessive fat production. This study aimed to identify candidate genes and pathways associated with intramuscular fat deposition on highly divergent marbling phenotypes in adult Hanwoo cattle.

**Results:**

Bovine genome array analysis was conducted to detect differentially expressed genes (DEGs) in *m. longissimus *with divergent marbling phenotype (marbling score 2 to 7). Three data-processing methods (MAS5.0, GCRMA and RMA) were used to test for differential expression (DE). Statistical analysis identified 21 significant transcripts from at least two data-processing methods *(P < 0.01)*. All 21 differentially expressed genes were validated by real-time PCR. Results showed a high concordance in the gene expression fold change between the microarrays and the real time PCR data. Gene Ontology (GO) and pathway analysis demonstrated that some genes (*ADAMTS4, CYP51A *and *SQLE*) over expressed in high marbled animals are involved in a protein catabolic process and a cholesterol biosynthesis process. In addition, pathway analysis also revealed that *ADAMTS4 *is activated by three regulators (*IL-17A*, *TNFα *and *TGFβ1*). QRT-PCR was used to investigate gene expression of these regulators in muscle with divergent intramuscular fat contents. The results demonstrate that *ADAMTS4 *and *TGFβ1 *are associated with increasing marbling fat. An *ADAMTS4/TGFβ1 *pathway seems to be associated with the phenotypic differences between high and low marbled groups.

**Conclusions:**

Marbling differences are possibly a function of complex signaling pathway interactions between muscle and fat. These results suggest that *ADAMTS4*, which is involved in connective tissue degradation, could play a role in an important biological pathway for building up marbling in cattle. Moreover, *ADAMTS4 and TGFβ*1could potentially be used as an early biological marker for marbling fat content in the early stages of growth.

## Background

Intramuscular fat deposition in cattle starts to become visible at 12 months of age and the rate of deposition increases from 15 months to 24 months [1]. The initial formation of visible intramuscular fat seems to be driven through the development of adipocytes in combination with declining muscle growth [2]. It has been shown that marbling fat content is negatively correlated with protein content in beef muscle [3]. In addition the development of adipose tissues in longissimus muscle of high-marbled cattle appears to disorganize the structure of the intramuscular connective tissue during growth [1]. This suggests that there might be an interaction between fat development and collagen structure in muscle. Kokta *et al *[4] reviewed the interaction between myogenic cells and adipocytes to determine the rate and extent of myogenesis and adipogenesis during animal growth. Fat and muscle development are regulated by a number of complicated biological pathways which are related to adenoreceptor signaling [5], the cytokine signaling pathway [6] and a wide range of hormonal and transcriptional factors such as leptin [7], adiponectin [8] and insulin like growth factor protein families [9]. As such, this interaction between muscle and fat also reflects a biochemical signaling pathway within the muscle. Therefore, marbling differences might be a function of a series of complex interactions between biological pathways [10,11].

The completion of the bovine genome project provided a tool for genome-wide functional studies to understand the interactions of complex biochemical pathways involved in protein and fat synthesis. For example, Affymetrix produces an oligonucleotide Bovine Genome Array that allows genome wide global profiling of over 23,000 bovine transcripts simultaneously Microarray based gene expression analyses associated with beef meat quality have focused on detecting differentially expressed genes in different breeds [12] and different nutritional treatments [13]. However, there is no report on gene expression differences in muscle with divergent marbling phenotypes within breed [14].

Here we report the results of a study undertaken to identify the biochemical differences in *m. longissimus *with divergent marbling phenotypes. The objective of this study was to identify differentially expressed genes and their role in a signaling pathway in *m. longissimus *with a wide range of marbling phenotypes.

## Results

### Differentially expressed genes between high and low marbling muscle

To detect the relationship between differentially expressed genes with marbling score, samples from *m. longissimus *were taken from ten unrelated animals with the highest and lowest marbling score. It was recognized that given the divergent nature of the animals the relationship would be inflated above that found in a normally sampled population.

Table 1 shows the summary statistics for the ten animals used in this microarray analysis. Intramuscular fat content (IMF) values range from 4% to 32% in the chosen animals with a clear contrast between the high and low marbled groups. After hybridization with the Affymetrix bovine genome array, gene expression intensities were measured using three pre-processing methods: MAS5.0, RMA and GCRMA.

**Table 1:** Summary statistics for marbling score, intramuscular fat and protein percentage for the low and high divergent marbling groups used for the gene expression analysis

Groups	Animal	Marbling score (range 1-7)	IMF (%)	Protein (%)
Low	509	2	7.11	21.07
	537	2	6.02	21.66
	554	3	4.88	21.30
	670	3	7.36	21.05
	691	3	12.04	21.23

High	527	7	24.35	16.06
	547	7	32.49	15.96
	586	7	16.56	17.27
	589	7	26.24	17.22
	632	7	18.81	18.07

Table 2 shows the summary of annotated probe sets on the bovine genome array. The array contains 24,128 probe sets, 91% of which were annotated by the manufacturer. Out of these, 12,745 (50%) were consistently detected as "Present (P)" in all samples according to the MAS5.0 background correction algorithm. Of the 12,745 probes in the P set, 12,184 have well annotated information (Table 2).

**Table 2:** Summary of annotated probes on the bovine genome array

Gene annotation	No of Genes in whole set: 24,128	No of Genes in present set: 12,745
Unidentified probe set (unknown genes)	2170	561
Annotated probe set	21958	12184
Bovine gene transcript annotated by Bovine genome sequence	9897 (45.07%)	5677 (46.59%)
Predicted genes defined by similarity in the bovine species	7275 (33.13%)	4232 (34.73%)
Transcripts defined by similarity in other species (human and mice)	4786 (21.79%)	2275 (18.67%)

**Figure 1:**
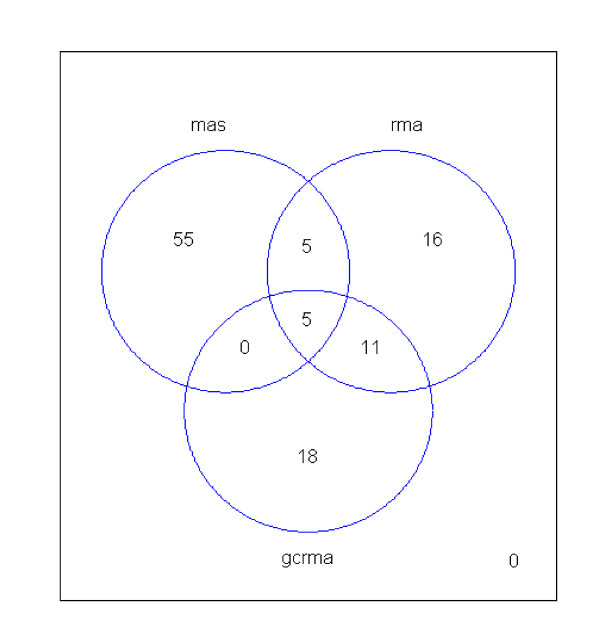
**Distribution of 136 differentially expressed genes across three different data-processing methods (MAS5.0; 65, RMA; 37 and GCRMA; 34). **The 21 differentially expressed genes were defined as those detected in at least two data processing methods.

A moderated t-test using Limma (33) was used to explore genes differentially expressed between high and low marbled animals in *m. longissimus*., We found 136 differentially expressed genes between high and low-marbled muscle using three data processing methods: MAS5.0 (65 transcripts), RMA (37 transcripts) and GCRMA (28 transcripts) (Figure 1).

**Figure 2:**
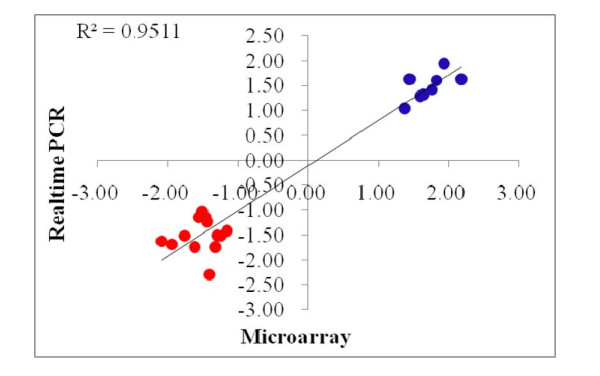
Technical validation for microarray results using realtime PCR: Correlation of fold changes between microarray and realtime PCR.

Twenty one genes showed significant differential expression (*P < 0.01*) in at least two normalization methods (Table 3). Out of the 21 differentially expressed genes (DEGs) listed in Table 3, eight differentially expressed genes were identified as up-regulated in muscle with high intramuscular fat content and the remaining 13 DEGs were down-regulated in samples with high intramuscular fat content. Putative genes were assigned to 14 out of the 21 DEGs (Table 3). The remaining 7 DEGs match gene sequences in bovine and other species with strong, moderate or weak sequence similarity but have no functional annotation assigned (Table 3). Based on the gene identities and associated functions, three up-regulated genes are involved in lipid metabolism (*squalene epoxidase *and *cytochrome P450*) and muscle metabolism (*SH3 domain YSC-like 1*). Three down-regulated genes (*ATP binding protein, Proteasome activator subunit 4 *and *Thimetoligopeptidase 1*) belong to functional classes involved in energy metabolism and an intracellular metabolic pathway. The remaining 7 DEGs are only hypothetical proteins or transcribed loci (single EST clones). Out of these 7 DEGs, three hypothetical proteins (*LOC788205, LOC509649 *and *LOC777601*) and two transcribed loci (Bt.19107.2.A1_at and Bt.19107.1.S1) could be worth pursuing further to elucidate their functional role in marbling. Technical validation of the 21 differentially expressed genes from the microarray experiment was confirmed by real-time PCR. The correlation of fold changes in gene expression between the arrays and PCR is shown in Figure 2. The results demonstrate a consistent gene expression pattern between both methods.

**Table 3:** The 21 differentially expressed genes in *m. longissimus *between high and low marbled Hanwoo

Probe ID	^**1**^**Gene Names**	^**2**^**Fold Change**				^**3**^**P-value (Modified F-test)**		
		
		MAS5.0	RMA	GCRMA	Mean	MAS5.0	RMA	GCRMA
Bt.5323.1.S1_at	SH3 domain YSC-like 1 (SH3YL1)	0.855	0.738	0.863	0.818	0.0006	0.0002	0.0005
Bt.15675.1.S1_at	ADAM metallopeptidase with thrombospondin type 1 motif, 4 (ADAMTS4)	0.938	0.822	1.121	0.953	0.0037	0.0003	0.0005
Bt.21021.1.S1_at	TBC1 domain family, member 7 (TBC1D7)	0.614	0.543	0.981	0.712	0.0084	0.0028	0.0017
Bt.2933.1.S1_at	Hypothetical protein LOC788205	0.982	0.434	0.591	0.668	0.0013	0.0015	0.0047
Bt.9767.1.S1_a_at	Squalene epoxidase (SQLE)	0.764	0.729	1.115	0.867	0.0048	0.0001	0.00002
Bt.621.1.S1_at	Cytochrome P450, family 51, subfamily A (CYP51A)	0.603	0.503	0.469	0.525	0.0080	0.0081	0.0178
Bt.23903.1.A1_at	Unknown	-0.948	-0.343	-0.299	-0.532	0.0087	0.0062	0.0232
Bt.22362.1.S1_at	SH3-domain kinase binding protein 1 (SH3KBP1)	-0.903	-0.829	-1.152	-0.960	0.0089	0.0090	0.0231
Bt.16752.1.A1_at	ATP binding protein (TXNDC9)	-0.805	-0.595	-0.679	-0.693	0.0123	0.0051	0.0006
Bt.1020.1.S1_at	CDC-like kinase 1 (CDClk1)	-0.398	-0.376	-0.451	-0.408	0.0231	0.0018	0.00005
Bt.19107.2.A1_at	Transcribed locus	-0.432	-0.583	-0.631	-0.548	0.0321	0.0024	0.0012
Bt.28011.1.S1_at	Unknown	-0.781	-1.212	-1.213	-1.066	0.0376	0.0022	0.0051
Bt.22718.1.A1_at	Proteasome (prosome, macropain) activator subunit 4 (PSME4)	-0.309	-0.312	-0.357	-0.326	0.0395	0.0087	0.0048
Bt.19107.1.S1_at	Transcribed locus	-0.515	-0.598	-0.813	-0.642	0.0729	0.0012	0.0045
Bt.25102.1.S1_a_at	Hypothetical LOC509649	-0.431	-0.515	-0.544	-0.496	0.0824	0.0033	0.0046
Bt.22038.1.S1_a_at	Arginyl-tRNAsynthetase (RARs)	-0.194	-0.238	-0.213	-0.215	0.1321	0.0055	0.0045
Bt.21268.1.S2_at	Ribosomal protein S6 kinase, 70 kDa (TUBD1)	0.263	0.471	0.643	0.459	0.1632	0.0045	0.0012
Bt.13342.1.S1_at	Src-associated protein SAW (UTP15)	-0.133	-0.462	-0.555	-0.383	0.2981	0.0011	0.0004
Bt.344.1.S1_at	Major histocompatibility complex, class II (BOLA-DMA)	-0.168	-0.537	-1.081	-0.595	0.3762	0.0069	0.0035
Bt.21827.2.S1_at	Thimetoligopeptidase 1 (THOP1)	-1.071	-0.531	-0.856	-0.818	0.0045	0.0087	0.0162
Bt.21794.1.S1_at	Hypothetical protein LOC777601	1.241	0.863	1.272	1.124	0.0046	0.0050	0.0149

^1^The 21 differentially expressed genes were selected from genes that were significant in at least 2 probe level summarization methods.

^2^Fold changes are shown on a log2 scale. Positive values show up regulation in high marbled animals and conversely, negative values show down regulation.

^3^P-value is set to P = 0.01 in this study.

### Gene Ontology (GO) and pathway analysis

DE genes were analyzed in the context of their GO biological process. Due to the incomplete annotation of the bovine genome, only 14 out of 21 differentially expressed probe sets were annotated. Top ranking biological GO terms are listed in Table 4, together with the genes associated to the GO term.

**Table 4:** Top-ranking GO biological process terms for genes DE between high and low marbled muscles

GO term/Affymetrix probe identifier	Entrez gene accession no	Gene Symbol	Gene description
Gene more highly expressed in high marbled muscle			
Protein kinase cascade:GO0007243 Bt.21268.1.S2_at	404181	RPS6KB1	Ribosomal protein S6 kinase, 70 kDa, polypeptide 1
Protein catabolic process:GO0006516			
Bt.15675.1.S1_at	286806	ADAMTS4	ADAM metallopeptidase with thrombospondin type 1 motif, 4
Germ cell development:GO0007281 Bt.21268.1.S2_at	404181	RPS6KB1	Ribosomal protein S6 kinase, 70 kDa, polypeptide 1
Cholesterol biosynthetic process:GO0006695			
Bt.621.1.S1_at	505060	CYP51	Cytochrome P450, family 51, subfamily A polypeptide 1
Regulation of Rab GTPase activity:GO0032313			
Bt.21021.1.S1_at	532704	TBC1D7	TBC1 domain family, member 7
Gene more highly expressed in low marbled muscle			
Protein kinase cascade:GO0007243 Bt.21827.2.S1_at	510889	THOP1	Thimet oligopeptidase 1
Peptide metabolic process:GO0006518			
Bt.21827.2.S1_at	510889	THOP1	Thimet oligopeptidase 1

**Figure 3:**
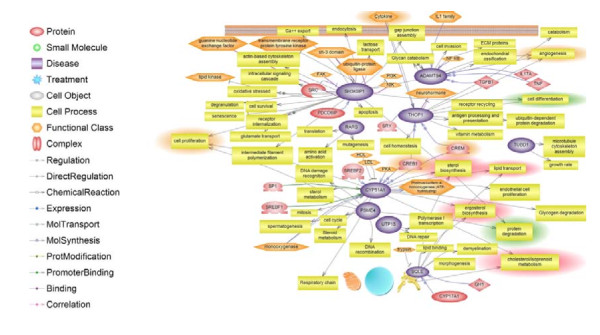
**Pathway studio analysis of 21 differentially expressed genes. **Out of 14 annotated genes, 9 genes were found to be involved in direct interactions. Five main pathway "hubs" (*SH3KBP1, ADAMTS4, CYP51A THOP1 and SQLE*) were detected in this pathway analysis. Each arrow indicates interactions between genes and a cell process pathway.

The pathway Studio v6.0 program (Ariadne Genomics, Inc) was used to identify molecular connections between the proteins encoded by the 14 annotated differentially expressed genes. The program searches through the ResNet database for all known interactions between genes/proteins such as physical interactions and regulation of expression (Figure 3). Out of the 14 annotated genes, 5 main pathway "hubs" (*SH3KBP1, THOP1, ADAMTS4, CYP51A *and *SQLE*) were detected in the pathway analysis. The *CYP51A *and *SQLE *proteins that are up-regulated in highly marbled muscle appear to be involved in steroid biosynthesis and cholesterol metabolism. In particular, *CYP51A *is activated by two proteins, *SP1 *and *SREBP1 *which are common transcription factors in lipid metabolism. *SH3KBP1 *is involved in cell processes such as intracellular signaling cascade, oxidative stress and cell proliferation. In addition, the pathway analysis demonstrated that *ADAMTS4 *is activated by immune responses related to single molecules (*IL-17A, TNF, NF-kB *and *IL-1 *family) and *transforming growth factor beta 1 *(*TGFβ1*). The results suggest a biological pathway connecting *CYP51A*, *SQLE *and *ADAMTS4 *that has not been previously identified in bovine gene expression studies on marbling fat.

### Gene expression of selected genes in the *ADAMST4 *pathway

GO and pathway analysis showed that *ADAMTS4 *is involved in protein catabolic process (GO0006516) and it is mainly activated by the immune related single molecules *IL-17A*, *TNFα *and *TGFβ1*. The *ADAMTS4 *gene has a role in proteolysis degradation of the extracellular matrix (connective tissue) in muscle. Marbling fat accumulates in the connective tissue matrix in close proximity to blood vessels. These findings suggest that *ADAMTS4 *might be involved in a pathway associated to phenotypic differences of marbling fat in cattle.

**Figure 4:**
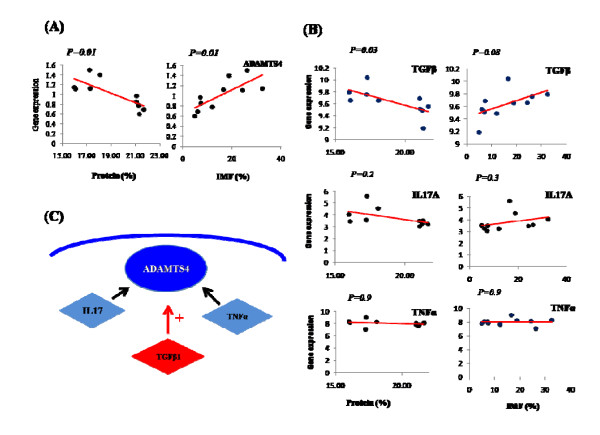
**Gene expression analysis of selected genes in *ADAMTS4 *pathway: **(A) gene expression for *ADAMTS4 *gene in muscle with divergent IMF and protein content. (B) gene expression for three stimulators (*IL17, TNFα and TGFβ1*) of *ADAMTS4 *protein in muscle with divergent IMF and protein content. (C) Co-expression of *ADAMTS4 *and *TGFβ1*.

To determine if any of the three regulators *IL-17A, TNFα *and *TGFβ1 *were associated with the *ADAMTS4 *pathway, we investigated gene expression of *ADAMTS4 *and the three regulators (*IL-17A, TNFα *and *TGFβ1*) in muscle with divergent IMF and protein content using RT-PCR. As shown in Figure 4, expression of *ADAMTS4 *increases significantly as intramuscular fat content increases (*P = 0.01*) and muscle protein content decreases (*P = 0.01*). Of the regulators only *TGFβ1 *significantly increased expression with increasing intramuscular fat content (*P = 0.03*) and it tended to decrease with decreasing muscle protein content (*P = 0.08*). In summary, *TGFβ1 *and *ADAMST4 *are highly associated with increases in marbling fat.

## Discussion

### Microarray analysis

A major objective of this study was to identify new biological indicators for marbling in cattle through global transcription profiling (24K Affymetrix chip). Identification of novel differentially expressed genes might allow a better understanding of the complex biochemical mechanisms of marbling in cattle. To date, microarray based gene expression analyses for beef meat quality (marbling) have focused on detecting differentially expressed genes in different breeds of cattle such as Japanese Black (Wagyu) and Holstein Friesian cattle for a wide range of marbling phenotypes [12,15]. As expected, many lipid metabolism related genes (for example, *FABP4 *and *SCD*) were highly expressed in the Japanese Black. As Pethick [2] commented, visible intramuscular fat content is a late maturing trait in cattle. Therefore, this study attempted to investigate genes that are differentially expressed in divergent marbling phenotypes at late stages of cattle growth.

So far, a well-known biochemical finding in relation to marbling expression in muscle was presented by Jurie *et al*. [16], who repored that *fatty acid binding protein 4 *activity was strongly correlated with intramuscular fat content. In their study they compared the different genetic performance of intramuscular fat deposition between two muscle types across three breeds. In this study however, fat metabolism related genes such as *FABP4, SCD *and *LPL *in muscle with divergent marbling phenotypes in late stage cattle were not detected. A similar study done by Liu *et al*.[17] in pigs indicated that FABP4 was differentially expressed among muscle samples with a divergent intramuscular fat content at 70 kg but wasn't different at a later stage (110 kg body weight). These findings suggest that many lipogenic related genes, including FABP4, will be more active during early growth during which IMF deposition is more intense, rather than at later growth stages. Alternatively, FABP4 is not detected as differentially expressed because all intramuscular fat was removed from the muscle samples used in this study (see details in the methods section).

In our study, all 21 differentially expressed genes were validated by real-time PCR. As shown in Figure 2, both methods showed consistent gene expression fold changes between the high and low marbled groups.

### Functional annotation of DEGs

Gene Ontology analysis and biological pathway searches were used to explore the functional annotation of the differentially expressed genes. One of the DEGs, *CDC like kinase 1(CLK1) *showed a negative correlation with increase in IMF content. This gene is part of the cell cycle signaling pathway. Using a rat model, Xiao *et al*. 2004 found that the *CLK *gene was also down-regulated with muscle fat during lactation. This might be due to decreasing expression of genes involved in protein synthesis through the cell cycle signaling pathway during lactation. Another gene encoding a *SH3 domainYSC-like 1 *was also up-regulated in animals with high IMF content. The *Src homology 3 (SH3) *domain is a small protein domain of about 60 amino-acid residues which was first identified as a conserved sequence in the non-catalytic part of several cytoplasmic protein tyrosine kinases. The function of the SH3 domain is not well understood. It seems to mediate the assembly of specific protein complexes via binding to proline-rich peptides. SH3 domain containing genes, sorbin and SH3 domain containing 1 (*SORBS1*), which are involved in insulin-stimulated glucose uptake [18] have also been reported as up-regulated in muscle of highly marbled Japanese Black Cattle (Wagyu) [19].

Gene Ontology analysis demonstrated that several genes that were more highly expressed in high marbled muscle are involved in protein catabolic and cholesterol biosynthesis processes. This was also reflected in the pathway analysis, which generated five major pathway "hubs" (*SH3KBP1, THOP1, ADAMTS4, CYP51A *and *SQLE*) for the genes more highly expressed in high marbled muscle. *CYP51A *and *SQLE *protein appear to be involved in steroid biosynthesis and cholesterol metabolism. In particular, *CYP51A *is activated by two transcriptional factors, *SP1 *and *SREBP1 *in lipid metabolism. More recently, Chen *et al*. [20] investigated gene expression of the *sterol regulatory element binding transcription factor 1 (SREBP1) *in muscle from differing sexes (female and male) and differing genotype within *SREBP1 *gene in pig. This study showed that the *SREBP1 *gene was highly expressed in muscle from female compared with males. In addition there were differences in expression within the genotypes showing a strong positive correlation with intramuscular fat content. The *CYP51A *pathway which is driven by the *SREBP1 *transcription factor might be one of the biological pathways associated to intramuscular fat in cattle. In this study SREBP1 was not differentially expressed in the arrays.

### Pathway for the *ADAMTS4 *gene

Marbling fat accumulates in a connective tissue matrix in close proximity to a blood capillary network between the bundle of muscle fibres in bovine skeletal muscle [21]. Under electron microscopy, reorganization and degradation of intramuscular connective tissue is observed in highly marbled muscle [1]. The relationship between marbling fat and protein content showed a negative correlation in *longissimus *of cattle; for example, *longissimus *with high level of fat decreased composition of moisture and protein in the carcasses [3]. Microarray analysis identified that *ADAMTS4 *is highly expressed in highly marbling muscle. *ADAMTS4 *has a function of metalopetidase that degrades the extracellular matrix of connective tissue [22]. Recently, one gene of the ADAM gene family, *ADAM12 was *reported as overexpressed in transgenic mice exhibiting increased intramuscular adipogenesis [23]. These findings suggest that *ADAMTS4 *might be one of the key genes controlling the relationship between marbling fat deposition and connective tissue degradation through a complex biological pathway in skeletal muscle.

**Figure 5:**
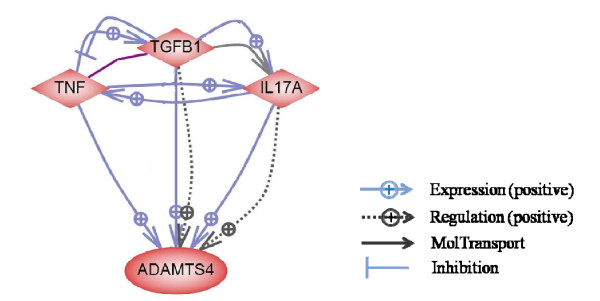
***ADAMTS4 *pathway. **The blue solid arrow with square indicates that the regulator changes the gene expression of the target gene. The dot arrow with square indicates that the regulator changes the protein activity of the target gene. The black solid arrow indicates that the regulator changes the localization of the target gene. The blue closed line indicates that the regulator inhibits gene expression of the target gene.

Pathway analysis (Figure 5) suggested that *ADAMTS4 *is activated by three regulators; *IL-17A, TNFα *and *transforming growth factor β 1 (TGFβ1) *that have not been previously considered to be associated with marbling fat. While only ADAMTS4 was detected as differentially expressed in the arrays (TNFα was excluded in the quality control step), the RT-PCR gene expression analysis found that *ADAMTS4 *and *TGFβ1 *are highly co-expressed in highly marbled muscle while these two genes have a lower expression with higher protein content (Figure 4). The *TGFβ1 *is known to be a member of the *GDF8 *family that is a major gene known to affect carcass fatness and double muscling in cattle [24]. The *GDF8 *gene not only affects the size of muscle but also the proportion of connective tissue within the muscle and intramuscular fat % [19]. Also, the *GDF8 *gene product is a growth regulator for muscle development. In cattle the mutation of this gene product causes a decrease in fat deposition and an increase in muscle mass of carcasses [25,26]. However, this study only shows that the *ADAMTS4/TGFβ1 *pathway might be involved in phenotypic differences between high and low marbled cattle. Therefore, an *ADAMTS4/TGFβ1 *pathway could be an important biological pathway related to increase of marbling fat in bovine skeletal muscle.

## Conclusion

Genome wide microarray analysis was undertaken to explore novel biological pathways associated with marbling in Hanwoo (*Korean cattle*). In this study, microarray analysis identified 21 differentially expressed genes (DEGs) in muscle with divergent marbling phenotypes. Pathway analysis for the 21 DEGs showed 5 unique pathway hubs associated with steroid biosynthesis, cholesterol metabolism and common transcription factors in lipid metabolism. These biological pathways might represent a phenomenon occurring in muscle with highly divergent marbling phenotypes. Out of these 5 main pathways, *ADAMTS4 *is involved in protein catabolic process (GO0006516), which is biologically related to the connective tissue degradation that is observed in highly marbled muscle. Pathway analysis revealed that *ADAMTS4 *gene is activated by three regulators; *IL-17A*, *TNFα*, and *transforming growth factor β 1 (TGFβ1) *that have not previously been considered to be associated with marbling fat. Gene expression analysis found that *ADAMTS4 *and *TGFβ1 *are co-expressed in highly marbled muscle while these two genes have a lower expression with higher protein content. We conclude *ADAMTS4 *might be one of the key genes controlling the relationship between marbling fat deposition and connective tissue degradation through a complex biological pathway in skeletal muscle. Further studies will be necessary to unveil the biological function of this pathway (*ADAMTS4/TGFβ1*).

## Methods

### Animals and sampling

The two divergent groups were formed by selecting the 5 highest and the 5 lowest marbling score carcasses from a population of 90 steers of pure bred Hanwoo cattle aged between 28 and 30 months old raised under similar conditions. Intramuscular fat (IMF %) of the muscle samples were measured using chemical fat extraction procedures and crude protein was measured on extracted samples (1 to 1.5 g) using the Macro-Kjeldahl method [27]. Table 1 presents summary statistics for all muscle samples used in this study.

### Total RNA isolation and Microarray hybridization

From each animal, after removal of intramuscular fat from the samples, total RNA samples were prepared from 1 g of frozen m. *longissimus *tissue using Trizol reagent (Invitrogen Inc., USA) according to the manufacturer's instructions. Messenger RNA was isolated from 500 μg of total RNA using Qiagen Oligotex resin (Qiagen Inc, USA). The quantity and quality of RNA samples were measured using absorbance at 260 nm and 280 nm in a capillary type spectrophotometer (Agilent Inc, USA) and confirmed in 1.2% formaldehyde-contained gel electrophoresis.

### Target preparation and high-density array hybridization

Double stranded cDNA was synthesized from 3 μg mRNA using a Genechip Expression 3'-Amplification One Cycle Synthesis kit (Affymetrix Inc. USA). The cDNA was purified using a Genechip Sample Cleanup Module (Affymetrix Inc). Biotin-labeled cRNA was synthesized in vitro using the Gene chip Expression 3'-Amplification reagents in the IVT labeling kit (Affymetrix Inc.). Biotin-labeled antisense cRNA was purified using the Genechip Sample Cleanup Module (Affymetrix Inc.) and the cDNA was fragmented in the 5 × Fragmentation buffer provided with the Genechip Sample Cleanup Module (Affymetrix Inc). Hybridization cocktail (200 μl) containing 15 μg fragmented cRNA was injected into the Genechip Bovine Genome Array (Affymetrix Inc). The array was placed in a 45 degrees hybridization oven at 60 rpm for 16 hours. After hybridization, the arrays were washed and stained in a fluidic station with the appropriate signal amplification protocol using biotinylated anti-streptavidin antibody (Vector Laboratories Inc., USA) and phycoerythrin-conjugated streptavidin (Invitrogen Inc., Carlsbad, CA, USA). The array was scanned with a GeneChipScanner 3000 (Affymetrix Inc.).

### Microarray Data processing

All quality control measures, preprocessing and analyses were performed using the statistical computing language R [28]. The quality of the arrays was assessed through standard quality control measures for Affymetrix arrays: pseudoimages of the arrays, MA scatter plots of the arrays versus a pseudomedian reference chip and other summary statistics including histograms and box plots of raw log intensities, box plots of relative log expressions, box plots of normalized unscaled standard errors and RNA degradation plots [29]. Transcription intensities in log2 scale were estimated from the probe-level data by using three summarization methods: MAS5.0 [30], RMA [30] and GCRMA [31]. In MAS5.0, each probe was adjusted using a weighted average. All arrays were scaled to the same mean value for normalization (200) and were summarized by a log2 scale average using 1-step Tukey biweight. For RMA, the background was corrected by convolution. The data were quantile normalized and summarized by median polish. GCRMA background correction used an affinity measure model based on probe sequences and mismatch intensities. Presence calls [32] for the probes were also calculated (τ = 0.015, α1 = 0.04 and α2 = 0.06). The data were filtered to remove control probes (n = 133) and probes detected as marginal or absent in all arrays using MAS5 presence calls.

### Microarray data analysis

Prior to testing for differential expression, the data were filtered to remove Affymetrix control probes (n = 133) and all noninformative probes detected as marginal or absent in all arrays (n = 11,383), thus remaining 12,745 probes to be tested. Differential transcription was tested for each summarization method using LIMMA [33,34]. Only differentially expressed (DE) probes detected in two out of the three summarization methods (*P < 0.01*) and flagged as present in at least 50% of the samples were considered to be significant. This approach ensures maximum specificity to detect differential expression and minimizes the effect of different summarization methods which are the main source of variability in the analysis of Affymetrix arrays. No false discovery rate correction method is warranted due to the stringency of the filtering criteria [35].

### QRT-PCR for the 21 DEGs and selected genes from the *ADAMTS4 *pathway

For technical validation of the microarray results, the 21 differentially expressed genes were validated by quantitative realtime RT-PCR (qRT-PCR). cDNA synthesis was performed using 2 μg of total RNA. The complimentary strand was primed with a random primer (Promega.co) and cDNA synthesis was performed using a Superscript III kit (Invitrogen) according to the manufacturer's instructions. Each quantitative PCR was carried out in a final volume of 20 containing 1 μl cDNA (500 ng/μl), 2 × SYBR Green I Master Mix (10 μl) (Qiagen., GmbH, Germany), and 10 pM forward and reverse primers. The real-time PCR reactions started at 95°C for 15 min for pre-denaturation and the condition was set at 95°C for 10 s, 56°C for 20 s and 72°C for 30 s. The PCR performed 40 cycles. The PCR was conducted in ABI 7500 realtime PCR system (Applied Biosystems, USA). Primer sets used in the real-time PCR are listed in Table 5.

**Table 5:** Primer sequences used in qRT-PCR

Probe ID	Gene Name	NCBI accession no.	Forward primer (5'->3')	Reverse primer (5->3')
Bt.5323.1.S1_at	SH3 domain YSC-like 1 (SH3YL1)	CB535095	ACCAACCCATAGAAGTGACAGCAC	CGAAGCTTTCCTTCCCACCAATCA
Bt.15675.1.S1_at	ADAM metallopeptidase with thrombospondin motif 4 (ADAMTS4)	NM_181667	AGTTCGACAAGTGCATGGTGTGTG	TGGTGACCACGTTGTTGTATCCGT
Bt.21021.1.S1_at	TBC1 domain family, member 7 (TBC1D7)	CB451394	CTTCGTGAACCAGCTGAACAGCAA	CGGCAAAGCACTTCTTGAACCACA
Bt.2933.1.S1_at	Hypothetical protein LOC788205	CK972377	GCCAAAGCAGCTGTCGGTAATGAA	TCCATCACACCGCGAAGACTCTAA
Bt.9767.1.S1_a_at	Squalene epoxidase (SQLE)	CK949309	AGTAATCATCGTGGGATCTGGCGT	ACCTGGGCATCAATACCTTCCACT
Bt.621.1.S1_at	Cytochrome P450, family 51, subfamily A (CYP51A)	BE664559	GTATGACCTCAACAACCCTGCCAA	TGACCACGACGATGATGAAGACCA
Bt.23903.1.A1_at	Unknown	BP102962	ACACAGGCCGTGCAAACTAAACAC	TCTTGATTTGCTGCTGGGACCTCT
Bt.22362.1.S1_at	SH3-domain kinase binding protein 1 (SH3KBP1)	CK774919	TGAGGGATGCACAGATGAGTGTGA	TTGAAGGCTGGAGGGCACATCTTT
Bt.16752.1.A1_at	ATP binding protein (TXNDC9)	CB536841	TTCATCTGCTGATGGCCACACTC	AGAGAATCCAGACTCTCCTCAGA
Bt.1020.1.S1_at	CDC-like kinase 1 (CDClk1)	CK848317	TTTAGGATGGTCCCAGCCATGTGA	GCCCAAGAATCCTTTCCATCATTGCC
Bt.19107.2.A1_at	Transcribed locus	BE723026	CCATGAGAACTGACTCGGGAGTTT	CAGGCTGTCTGGCAAGCAACAATA
Bt.28011.1.S1_at	Unknown	CK940528	AGGAAGAACCTTCTGTCCCAGCTT	TTGAGACTTCCCAGGTCAAAGGCA
Bt.22718.1.A1_at	Proteasome (prosome, macropain) activator subunit 4	CK945034	GCTGAGGTGTGGGTTTGTTTGAGT	AAACTGGTCACAGGCAAACACG
Bt.19107.1.S1_at	Transcribed locus	BE723026	GACCCAAGAGTTGCTTAAGAGAGC	ACCCTCAGTCCACAGATGATCAAG
Bt.25102.1.S1_a_at	Hypothetical LOC509649	CK772143	TTCCTCCCACTGGTGAGCATCTTT	TGTGTTGCTCAGTGTTCTCCTCCA
Bt.22038.1.S1_a_at	Arginyl-tRNAsynthetase (RARs)	CK947459	TTGAAGGCTGCTCAGACCTCTGTT	TAAGCCGCTGTGTTTCCTCTGTCA
Bt.21268.1.S2_at	Ribosomal protein S6 kinase, 70kDa (TUBD1)	CK977623	CGTGACTGTAGATGGTGAAAGGGT	TGCACACTCAGACTGAAGACAC
Bt.13342.1.S1_at	Src-associated protein SAW (UTP15)	BM030756	CTCATAGCCATCAATAGTTCAGTGC	TCAAGTAGCAAATACTACAGTTTGTC
Bt.344.1.S1_at	Major histocompatibility complex, class II	D76416.1	TCACACCAGCACCCTCTGATCTTT	TAAGCACGGCTTTCGGCAGTAGAA
Bt.21827.2.S1_at	Thimetoligopeptidase 1 (THOP1)	CB444022	AAGGTCTCCATCTGGAGGTGTTTG	AACTCCCAGGAAAGGGCTGCATT
Bt.21794.1.S1_at	Hypothetical protein LOC777601	CA034934	CATGAGACACAGGCGAAACACTGA	TCTTTGGGAGAAAGGGAAACTGGG
G3PDH	Glyceride 3 phosphatate dehydrogenase	AY779626	GGGTCATCATCTCTGCACCT	GGTCATAAGTCCCTCCACGA
IL17A	Interleukin 17A	CB432107	TCATCATCCCACAGAGTCCA	GGAGAGTCCAAGGTGAGGTG
TNFα	Tumor necrosis factor	CK848164	GGCCATGGTATTGACATCCT	GGATCTTCTCCACCACATCG
TGFβ1	Transforming growth factor beta 1	CK772652	ACTACATCTCGGCGCTCAGT	GAAGGTGCAGGTGAAGTGGT

The relative gene expression value was calculated by the ΔCt method [36]. The ΔCt value of the target gene was normalized against the *G3PDH *Ct value. The fold change was determined as 2^-ΔCt^.

For selected genes in the putative *ADAMTS4 *pathway, a regression analysis of gene expression value (2^-ΔCt^) on intramuscular fat and protein content was performed using a simple linear regression in R [28].

### Gene ontology (GO) analysis

Annotation of DE probes was performed using the Database for Annotation. Visualization and Integrated Discovery (DAVID) http://david.abcc.ncifcrf.gov/home.jsp. In subsequent text the term "probe" is replaced by "gene". The DE genes were analyzed in the context of their gene ontology (GO) biological process (Gene Ontology Consortium. 2006).

### Biological pathway analysis

Differentially expressed genes in the *m. longissimus *of high and low marbled Hanwoo were clustered into pathways using the program Pathway studio (Stratagene, La Jolla, CA). This program provided a visual representation of the differentially expressed genes from the microarray data. In this study, 14 annotated genes out the 21 differentially expressed genes were imported into the program. Pathway studio then generated relationships between gene products using a literature search of curated genes and displayed the interactions in the form of pathways that can also include other genes/proteins, small molecules, cellular processes, and relevant transcription factors. If no interactions were found or proper annotation was not available, the gene was not included in the pathway. To help explicit the function of the genes in each pathway, gene ontology terms were determined for all genes illustrated within the pathways generated by Pathway studio 6.0.

## Authors' contributions

S.H.L and C.G were involved in designing and planning of the study. They carried out the experiments and analyses, interpreted data, annotation and statistical analyses and wrote the first draft of the paper. N.K.K and D.L were involved in the realtime PCR analysis, pathway analysis and contributed to the writing of the paper. E.W.P and S.J.O was involved in the experimental design and contributed to the writing of the paper. S.J.O, J.V, J.P.G and J.M.T conceived the project, designed and carried out analysis, interpreted data, and were involved in the writing of the paper. All authors have read and approved the final manuscript.
